# 8‐Hydroxyquinolines are bactericidal against Mycobacterium tuberculosis

**DOI:** 10.1002/ddr.21531

**Published:** 2019-03-20

**Authors:** Joshua O. Odingo, Julie V. Early, Jake Smith, James Johnson, Mai A. Bailey, Megan Files, Junitta Guzman, Juliane Ollinger, Aaron Korkegian, Anuradha Kumar, Yulia Ovechkina, Tanya Parish

**Affiliations:** ^1^ TB Discovery Research Infectious Disease Research Institute Seattle Washington

**Keywords:** *Mycobacterium* tuberculosis, antibacterial, hydroxyquinoline, structure–activity relationship, *tuberculosis*

## Abstract

There is an urgent need for new treatments effective against Mycobacterium tuberculosis, the causative agent of tuberculosis. The 8‐hydroxyquinoline series is a privileged scaffold with anticancer, antifungal, and antibacterial activities. We conducted a structure–activity relationship study of the series regarding its antitubercular activity using 26 analogs. The 8‐hydroxyquinolines showed good activity against *M. tuberculosis,* with minimum inhibitory concentrations (MIC90) of <5 μM for some analogs. Small substitutions at C5 resulted in the most potent activity. Substitutions at C2 generally decreased potency, although a sub‐family of 2‐styryl‐substituted analogs retained activity. Representative compounds demonstrated bactericidal activity against replicating *M. tuberculosis* with >4 log kill at 10× MIC over 14 days. The majority of the compounds demonstrated cytotoxicity (IC_50_ of <100 μM). Further development of this series as antitubercular agents should address the cytotoxicity liability. However, the 8‐hydroxyquinoline series represents a useful tool for chemical genomics to identify novel targets in *M. tuberculosis*.

## 1 INTRODUCTION

Tuberculosis is the leading killer among infectious diseases with 1.3 million deaths and >10 million new cases in 2017 (World Health Organization, 2018). The shortest treatment for tuberculosis is a 6‐month regimen containing four antibiotics (World Health Organization, 2018). More than half a million multidrug resistant (MDR) cases emerge each year, with treatment of those cases requiring up to 2 years of treatment with second and third line drugs (World Health Organization, 2018). Affordable and innovative treatments with shorter treatment times and unique mechanisms of action are urgently needed.

8‐hydroxyquinoline (HQ) is a well‐studied, privileged structure, with activity against a wide range of cell types (Prachayasittikul, Prachayasittikul, Ruchirawat, & Prachayasittikul, 2013; Song, Xu, Chen, Zhan, & Liu, 2015), including anticancer activity (Barilli et al., 2014; Bhat, Shim, Zhang, Chong, & Liu, 2012; Chang, Chen, Wang, Tzeng, & Chen, 2010; Feng et al., 2015; Li et al., 2016; Moret et al., 2009; Mrozek‐Wilczkiewicz et al., 2015; Sosič et al., 2013; Spaczyńska, Tabak, Malarz, & Musiol, 2014), antifungal activity (Cieslik et al., 2012; Musiol et al., 2006), and antibacterial activity (Warner, Musto, Turesky, & Soloway, 1975). The HQ are active against several bacterial species including *Staphylococcus aureus* and *Staphylococcus epidermidis* (Abouelhassan et al., 2014, 2015; Basak et al., 2016; Garrison et al., 2017; Lam et al., 2014), *Enterococcus faecium* (Basak et al., 2016; Garrison et al., 2017), *Burkholderia pseudomallei* (Wangtrakuldee et al., 2013), *Neisseria gonorrhoeae,* (Lawung et al., 2018), *Listeria monocytogenes* (Cherdtrakulkiat et al., 2016), and *Mycobacterium avium* (Hongmanee, Rukseree, Buabut, Somsri, & Palittapongarnpim, 2007; Kos et al., 2015). In addition, activity of the HQ compounds against replicating and nonreplicating *Mycobacterium tuberculosis* has been demonstrated in medium containing copper and in infected guinea pigs (Ananthan et al., 2009; Darby & Nathan, 2010; Hongmanee et al., 2007; Shah et al., 2016; Tison, 1952; Urbanski, Slopek, & Venulet, 1951). Despite these studies, the structure–activity relationship (SAR) of HQs for antitubercular activity has not been thoroughly explored. To establish a more complete understanding of the SAR of HQ regarding antitubercular activity, we conducted an in vitro study, focusing on growth inhibition of *M. tuberculosis* in standard culture medium using a set of 26 commercially available compounds containing HQ functionality.

## 2 METHODS

### 2.1 Chemicals

Chemicals were purchased from Chembridge Corp. (San Diego, CA), Sigma‐Aldrich Co. (St. Louis, MO), or ChemDiv Inc. (San Diego, CA). Compound purity was verified via LCMS on an Agilent 1100 series instrument (Phenomenex Luna C18, 4.8 mm × 150 mm, 1.0 mL/min, UV 254 nm, electrospray ioniziation) with MeCN/H_2_O (0.05% HCO_2_H or NH_4_OAc buffer) gradient elution.

### 2.2 Cytotoxicity

HepG2 human liver cells (ATCC) were seeded in 384‐well plates at 1,800 cells per well and incubated in a humidified atmosphere of 37°C, 5% CO_2_ in DMEM (Invitrogen), 10% FBS, 1 mM sodium pyruvate, 2 mM Glutagro (Corning), 25 mM glucose, 100 I.U/mL penicillin, and 100 μg/mL streptomycin. Compounds were added 24 hr post cell seeding to 1% DMSO final concentration. After a 72 hr incubation period, CellTiter‐Glo® Reagent was added to the 384‐well plates. Relative luminescent units (RLUs) were measured using a Biotek Synergy 4 plate reader. Raw data were normalized by the average RLU value from 1% DMSO treated wells and expressed as percent growth. Growth inhibition curves were fitted using the Levenberg–Marquardt algorithm. The IC_50_ was defined as the compound concentration that produced 50% of the growth inhibitory response.

Vero cells (CCL81) were cultured in high‐glucose Dulbecco's modified Eagle medium with GlutaMax (Invitrogen), 10% v/v fetal bovine serum, 50 units/mL penicillin, and 50 μg/mL streptomycin. Cells were exposed to compounds for 2 days and viability was determined using CellTiter‐Glo (Promega). The % viability was plotted and the concentration that resulted in 50% reduction in cell viability was determined using the Levenberg–Marquardt least‐squares method.

### 2.3 Determination of minimum inhibitory concentration

*M. tuberculosis* strains were grown in Middlebrook 7H9 supplemented with 10% v/v oleic acid, albumin, dextrose, and catalase (OADC) (Becton Dickinson) and 0.05% w/v Tween 80 or on Middlebrook 7H10 agar supplemented with 10% v/v OADC. MICs were determined as previously described (Ollinger et al., 2013). In brief, compounds were tested for activity at varying concentrations against a fluorescent strain of *M. tuberculosis* H37Rv constitutively expressing a red fluorescent protein (mCherry or *Ds*Red) (Carroll et al., 2010; Carroll, Muwanguzi‐Karugaba, & Parish, 2018), typically starting at 20 μM. Plates were incubated for 5 days and bacterial growth was measured using optical density and fluorescence. Growth was plotted, and the concentration required to inhibit growth by 90% was determined using the Levenberg–Marquardt least‐squares method (Lambert & Pearson, 2000).

### 2.4 Kill kinetics

Compounds **24** and **4** were tested for their ability to kill *M. tuberculosis* in replicating conditions (Early & Alling, 2015). Compounds were added to *M. tuberculosis* cultures in liquid medium and viability monitored over 3 weeks standing at 37°C by plating for colony‐forming units (CFU). Plates were incubated at 37°C for 4 weeks before colonies were counted.

## 3 RESULTS AND DISCUSSION

### 3.1 SAR studies

The HQ series has good activity against *M. tuberculosis* in vitro. We, and others, identified these compounds in phenotypic screening as hits (Ananthan et al., 2009; Ollinger et al., 2018). We wanted to explore the SAR for this series to determine the scope for further development. We designed a set of compounds representing three general chemotypes built around the HQ scaffold: (a) substituted at the 5‐position (Table 1), (b) substituted at the 2‐position (Table 2), and (c) styryl substituents at the 2‐position (Table 3). For each compound, we determined activity against *M. tuberculosis* by measuring the minimum inhibitory concentration (MIC_90_), defined as the concentration required to inhibit growth by 90%. We also determined cytotoxicity against the HepG2 cell line by measuring the IC_50_, defined as the concentration required to reduce cell viability by 50%. The seed compound, unsubstituted 8‐hydroxyquinoline (**1**), had an MIC of 3.6 μM and HepG2 IC_50_ of 7.6 μM.

**Table 1:** Activity of 5‐substituted compounds

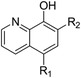					
Cpd	ID	*R*_1_	*R*_2_	MIC (μM)	HepG2 IC_50_ (μM)
1	IDR‐0261683	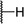	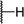	3.6 ± 0.20 (2)	7.6 ± 0.68 (4)
2	IDR‐0257179	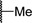	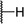	3.1 ± 1.1 (4)	6.5 ± 1.7 (2)
3	IDR‐0010414	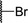	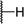	2.9 ± 0.40 (2)	6.9 ± 0.59 (4)
4	IDR‐0261686	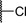	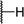	3.0 ± 1.0 (4)	7.6 ± 1.5 (2)
5	IDR‐0105904	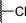	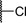	8.4 ± 4.5 (6)	7.6 ± 1.2 (4)
6	IDR‐0261687	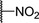	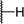	4.8 ± 3.3 (3)	7.7 ± 1.8 (2)
7	IDR‐0271627	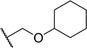	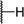	3.2 ± 0.85 (4)	4.9 ± 1.9 (2)
8	IDR‐0257187	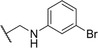	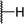	>20 (2)	12 ± 0.50 (2)
9	IDR‐0257186	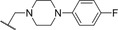	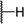	>20 (2)	9.8 ± 2.2 (2)
10	IDR‐0261684	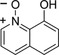		>20 (2)	<88 ± 17 (2)

Compounds were tested for activity against *M. tuberculosis* (MIC) and HepG2 cells (IC_50_). Data are the mean ± *SD*. The number of replicates is in parentheses.

**Table 2:** Activity of 2‐substituted compounds

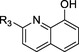				
Cpd	ID	R_3_	MIC (μM)	HepG2 IC_50_ (μM)
11	IDR‐0261685	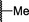	>20 (4)	28 ± 9.0 (2)
12	IDR‐0257175	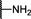	4.6 ± 1.7 (3)	12 ± 0.50 (2)
13	IDR‐0257176	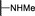	6.4 ± 0.35 (2)	22 ± 2.8 (2)
14	IDR‐0195922	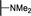	>20 (2)	>100 (2)
15	IDR‐0143082	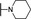	>20 (2)	>100 (2)
16	IDR‐0126687	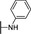	>20 (2)	3.8 ± 0.45 (2)
17	IDR‐0126637	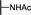	>20 (3)	9.0 ± 1.0 (2)
18	IDR‐0257178	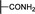	>20 (2)	>100 (2)
19	IDR‐0257177	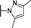	>20 (3)	12 ± 0.50 (2)

Compounds were tested for activity against *M. tuberculosis* (MIC) and HepG2 cells (IC_50_). Data are the mean ± *SD*. The number of replicates is in parentheses.

**Table 3:** Activity of vinylogously 2‐substituted compounds

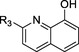				
Cpd	ID	*R*_3_	MIC (μM)	HepG2 IC_50_ (μM)
20	IDR‐0257190	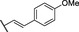	23 ± 7.0 (2)	12 ± 4.0 (2)
21	IDR‐0257183	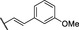	8.7 ± 0.2 (2)	10 ± 1.0 (2)
22	IDR‐0257182	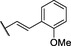	13 ± 9.1 (2)	12 ± 2.0 (2)
23	IDR‐0257180	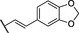	15 ± 5.0 (3)	9.1 ± 3.0 (2)
24	IDR‐0010378	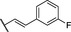	13 ± 6.3 (4)	8.0 ± 0.60 (2)
25	IDR‐0257181	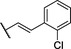	5.8 ± 2.2 (2)	12 ± 1.0 (2)
26	IDR‐0257188	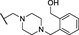	>20 (2)	14 ± 0.50 (2)

Compounds were tested for activity against *M. tuberculosis* (MIC) and HepG2 cells (IC_50_). Data are the mean ± *SD*. The number of replicates is in parentheses.

Our exploration began with the introduction of substituents at the 5‐position (Table 1). Compounds with methyl (**2**), bromide (**3**), chloride (**4**), and nitro (**6**) substituents had comparable potency and cytotoxicity to the parent (**1)**. Addition of a second chloride at the 2‐position (**5**) resulted in slightly decreased potency (8.4 μM). The larger (cyclohexyloxy)methyl group (**7**) retained both potency and cytotoxicity. However, introduction of aniline‐containing groups (**8** and **9**) abrogated the anti‐tubercular activity with only a minor reduction in cytotoxicity. Oxidation to the N‐oxide (**10**) resulted in loss of activity and a significant reduction of cytotoxicity, suggesting that the quinoline lone pair was necessary for both.

Of the 8‐hydroxyquinolines directly substituted at the 2‐position (Table 2), only the simplest amine substitutions **12** and **13** retained measurable anti‐tubercular activity. The region proved generally intolerant of larger amine substituents, with dimethylamine (**14**), 1‐piperidine (**15**), and aniline (**16**) substituents all resulting in loss of activity. Acylation of the exocyclic amine (**17**) and its reverse amide analog (**18**) were similarly not tolerated. Methyl substitution (**11**) was not tolerated. Analogs **14**, **15**, and **18** from this chemotype showed significant attenuation of mammalian cytotoxicity; unfortunately, all were inactive against *M. tuberculosis*.

Finally, the scaffold proved broadly tolerant to substitution with styryl groups at the 2‐position, albeit with the induction of significant variation between replicates (Table 3). Substitutions on the styryl ring (**20**–**25**) led to only minor changes in potency. As with the active compounds from the previous two chemotypes, these compounds were consistently cytotoxic. Replacement of the vinyl substructure with a piperazine (**26**) was not tolerated.

### 3.2 Kill kinetics

**Figure 1:**
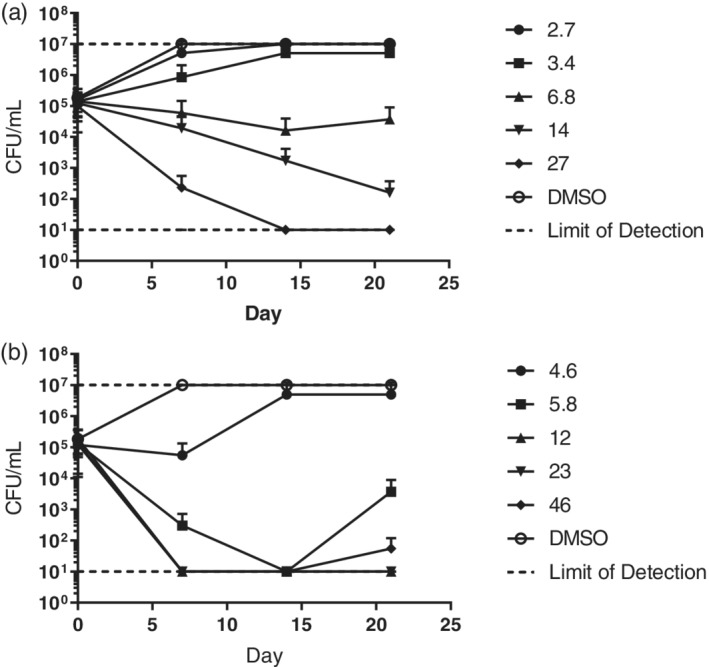
Bactericidal activity of compounds against replicating *M. tuberculosis*. Compounds (24) (top) and (4) (bottom) were tested for their ability to kill *M. tuberculosis* under replicating conditions. *M. tuberculosis* was cultured in 7H9‐OADC‐Tw medium in the presence of compounds from 1× MIC to 10× MIC (concentrations given in μM). Viable bacteria were counted by measuring CFUs. Data are the mean ± *SD* from two independent experiments

We determined the kill kinetics of two active compounds that were randomly selected against aerobically‐grown, actively replicating *M. tuberculosis*. Compounds **24** and **4** were both bactericidal against *M. tuberculosis* under replicating conditions (Figure 1). Compound **24** killed ≥2 logs of bacteria at concentrations above 14 μM (5× MIC). At 27 μM (10× MIC), the compound sterilized the culture (>4 log kill) within 14 days. Compound **4** killed ≥3 logs within 14 days at concentrations of 12 μM (2.5× MIC) and higher. These data are consistent with previous reports that this series demonstrates rapid kill kinetics in vitro (Darby & Nathan, 2010). For both compounds, a small increase in viable bacteria was seen at the lowest concentrations after 21 day (for **24** at 6.8 μM and for **4** at 5.8 μM). It is likely that this represents outgrowth of resistant mutants at the later time point. Alternatively, it could reflect compound instability over the extended exposure period, but that has not been tested.

### 3.3 Cytotoxicity

The main liability for this series is cytotoxicity. Three analogs (**14, 15, 18**) showed no measurable cytotoxicity against HepG2 cells, but none of these were active against *M. tuberculosis*. All 19 of the active compounds were cytotoxic for this cell line. To compare cell lines, all 26 analogs were tested for cytotoxicity against Vero cells; all active analogs were cytotoxic in this cell line as well (Table 4). These cytotoxicity data are in contrast to results from other groups, where cytotoxicity for 8‐hydroxyquinoline (**1)** and related compounds was not noted (Ananthan et al., 2009; Darby & Nathan, 2010; Shah et al., 2016). The differences in these data may reflect the status of cells in the cytotoxicity assay, since, similar to our findings, Shen et al. found HQ analogs to be cytotoxic via inhibition of DNA synthesis in HepG2 cells (Shen, Chen, & Roffler, 1999). Since our assay uses replicating HepG2 or Vero cells to measure toxicity, we would expect to see negative effects from the inhibition of DNA synthesis and by extension cell division. We were unable to identify analogs that lacked toxicity against replicating HepG2 or Vero cells. Further work to identify the mechanism of action of the HQ compounds, which may or may not involve metal chelation, might reveal a way forward in designing analogs with specificity for mycobacteria (Prachayasittikul et al., 2013; Shen et al., 1999).

**Table 4:** Cytotoxicity of HQ compounds

Compound	ID	Cytotoxicity (IC_50_)
**1**	IDR‐0261683	1.4
**2**	IDR‐0257179	1.4
**3**	IDR‐0010414	1.8
**4**	IDR‐0261686	2.4
**5**	IDR‐0105904	1.3
**6**	IDR‐0261687	2.9
**7**	IDR‐0271627	6.2
**8**	IDR‐0257187	7.5
**9**	IDR‐0257186	5.8
**10**	IDR‐0261684	>20
**11**	IDR‐0261685	12
**12**	IDR‐0257175	3.3
**13**	IDR‐0257176	8.2
**14**	IDR‐0195922	>50
**15**	IDR‐0143082	>50
**16**	IDR‐0126687	0.94
**17**	IDR‐0126637	4.9
**18**	IDR‐0257178	>50
**19**	IDR‐0257177	19
**20**	IDR‐0257190	1.6
**21**	IDR‐0257183	2.9
**22**	IDR‐0257182	6.2
**23**	IDR‐0257180	6.0
**24**	IDR‐0010378	7.2
**25**	IDR‐0257181	9.0
**26**	IDR‐0257188	11

Compounds were tested for activity against Vero cells (IC_50_).

## 4 CONCLUSION

In conclusion, we confirmed that the HQ series have good anti‐tubercular properties in vitro with rapid bactericidal activity. SAR demonstrated that small substitutions at the 5‐position were tolerated, but substitution at the 2‐position was not generally tolerated, except for 2‐styrenyl‐substitutions. Further work to elucidate the bacterial target could pave a way forward in designing analogs without cytotoxicity.

## CONFLICT OF INTEREST

The authors declare they have no conflict of interest.
